# Hypoxic stress disrupts HGF/Met signaling in human trophoblasts: implications for the pathogenesis of preeclampsia

**DOI:** 10.1186/s12929-022-00791-5

**Published:** 2022-02-03

**Authors:** Guanlin Li, Yongqing Wang, Guangming Cao, Yeling Ma, Yu-Xia Li, Yangyu Zhao, Xuan Shao, Yan-Ling Wang

**Affiliations:** 1 Clinical Stem Cell Research Center, Peking University Third Hospital, Beijing, China; 2 State Key Laboratory of Stem Cell and Reproductive Biology, Institute of Zoology, Institute for Stem Cell and Regeneration, Chinese Academy of Sciences, Beijing, China; 3 Beijing Institute for Stem Cell and Regenerative Medicine, Beijing, China; 4 Department of Obstetrics and Gynecology, Peking University Third Hospital, Beijing, China; 5 Department of Obstetrics and Gynecology, Beijing Chao-Yang Hospital, Beijing, China; 6 University of the Chinese Academy of Sciences, Beijing, China

**Keywords:** Preeclampsia, Met, Hypoxia, Endocytosis, Ubiquitin degradation, CAV-1, Cbl

## Abstract

**Background:**

Preeclampsia (PE), a placenta-associated pregnancy complication, is the leading cause of maternal and perinatal morbidity and mortality. Met/Erk signaling is inhibited in the placentas of patients with early-onset preeclampsia (E-PE), but the underlying mechanisms remain elusive. In this study, the expression modes of Met and endocytic vesicles in normal and preeclamptic placentas were compared. Biotinylation internalization/recycling assays were used to measure the endocytosis of Met under hypoxia and normoxia in HTR8/SVneo cells. In addition, the expression level of Cbl, a specific E3 ligase of Met, was measured under hypoxia and normoxia, and the endocytosis of Met was studied by using confocal microscopy.

**Results:**

We found considerable intracellular accumulation of Met, which was colocalized with caveolin-1 (CAV-1), in trophoblasts from E-PE placentas. Prolonged hypoxic stimulation led to the remarkable augmentation of CAV-1-mediated Met endocytosis in HTR8/SVneo cells. In addition, the expression of Cbl was substantially repressed by sustained hypoxia, disrupting ubiquitin degradation and the subsequent intracellular accumulation of Met in HTR8/SVneo cells. The abnormal degradation of Met hampered the ability of hepatocyte growth factor (HGF) to promote trophoblast cell invasion. In E-PE placentas, aberrant upregulation of CAV-1 and downregulation of Cbl were observed in parallel to the intracellular accumulation of Met.

**Conclusions:**

These findings reveal that prolonged hypoxic stress induces the augmentation of endocytosis and repression of ubiquitin-mediated Met degradation, which leads to the impaired regulation of trophoblast invasion by HGF/Met signaling. These data provide novel evidence for elucidating the pathogenesis of preeclampsia, especially of the early-onset subtype.

**Supplementary Information:**

The online version contains supplementary material available at 10.1186/s12929-022-00791-5.

## Background

Preeclampsia (PE), a leading cause of maternal and perinatal morbidity and mortality worldwide, is characterized by new-onset hypertension, proteinuria or dysfunction of multiple maternal organs after the 20th week of gestation [1–5]. Placental deficiencies are widely recognized as the principal pathological origin of the disorder.

Hepatocyte growth factor (HGF) is an essential regulator of placental development. Once Met binds to its ligand HGF, it is dimerized and phosphorylated, leading to its activation and stimulation of multiple downstream signaling pathways, including the MEK/Erk, phosphatidylinositol 3-kinase (PI-3K) and signal transduction and activators of transcription 3 (STAT3) pathways, which play important roles in the progression of trophoblast functions [6, 7]. Deficiency of HGF or its receptor Met in mice causes fetal lethality and placental dysfunction [8]. Multiple in vitro studies have demonstrated their involvement in placental cell events, including trophoblast migration, invasion and blood vessel remodeling [9, 10]. The Erk pathway, downstream of HGF/Met signaling, has significant roles in cell proliferation, cell survival and metastasis [11, 12]. Dysregulation of the Met/Erk pathway has been implicated in the preeclamptic placenta [13, 14]. Our previous investigation demonstrated the remarkable inhibition of Met/Erk signaling in the placentas of patients with early-onset PE (E-PE), which was partially attributed to the hampered transactivation of Met by Semaphorin4D (Sema4D) [15]. However, the mechanisms underlying aberrant Met/Erk signaling in E-PE placenta remain largely elusive.

Ligand-induced signaling through the membrane receptor is tightly controlled. One mechanism involves the rapid internalization and subsequent degradation or recycling of the receptor in a process called receptor-mediated endocytosis, via which the cells can quickly switch off further signaling to maintain appropriate receptor activation [7, 16]. Endocytic vesicles are 50–100 nm invaginations found within the plasma membrane of cells [17, 18]. Clathrin-mediated or caveolin-1 (CAV-1)-dependent endocytosis are the classic pathways involved in the downregulation of cell-surface receptors [19–22] and thus considered to be negative regulators of many signaling pathways [23–25]. It has been reported that hypoxia damages the internalization of membrane receptors, such as glutamate receptor 1 (GLR-1) and transferrin receptor [26–28], and PE placentas usually present with pathological hypoxia [29–31]. Moreover, evidence has confirmed the association of dysregulated ubiquitin-associated degradation with the onset of PE [32, 33]. Therefore, it is reasonable to speculate that hypoxia may disrupt the recycling and degradation of Met in PE placentas. However, the mechanism of Met endocytosis and degradation has not yet been clarified.

This evidence suggests that the impaired Met/Erk signaling in the E-PE placenta is due to abnormalities in the endocytosis and proteasomal degradation of Met in trophoblast cells. We tested this hypothesis by comparably analyzing the distributions and expression of Met, Clathrin, CAV-1 and Cbl in the placentas of PE patients and normal pregnant women. The CAV-1-mediated endocytosis and Cbl-coupled degradation of Met in human trophoblasts were examined under hypoxic stress. These findings reveal the mechanism by which sustained hypoxic stress induces the augmentation of endocytosis and repression of ubiquitin-mediated Met degradation and thereby hampers HGF/Met signaling to regulate trophoblast invasion. These data provide novel evidence for elucidating the pathogenesis of PE, especially the early-onset subtype.

## Methods

### Tissue preparation

Human placental tissues were collected from the Department of Obstetrics and Gynecology, Peking University Third Hospital (Beijing, China). The study protocol for tissue collection was approved by the Ethics Committee of Peking University Third Hospital and the Institute of Zoology, Chinese Academy of Sciences. Written informed consent was obtained from the enrolled pregnant women.

In brief, placental tissues (chorionic villi and decidua) were obtained from patients who underwent therapeutic termination of pregnancy at gestational weeks 6–11. Placental specimens from PE patients and normal pregnant women were collected following cesarean section. According to the definition in Williams Obstetrics (23rd edition) and the American College of Obstetricians and Gynecologists (ACOG) guidelines [34], severe PE was defined as a pregnancy having no history of preexisting or chronic hypertension but showing a systolic blood pressure ≥ 160 mmHg or a diastolic blood pressure ≥ 110 mmHg on at least 2 occasions, accompanied by significant proteinuria (≥ 2 g/24 h or 3 + by dipstick in two random samples collected at > 4-h intervals) or problems in multiple organs (such as pulmonary edema, seizures, oliguria, abnormal liver enzymes associated with persistent epigastric or right upper-quadrant pain, and persistent and severe central nervous system symptoms) after the 20th week of gestation. According to whether the onset time of clinical signs was earlier than the 34th week, PE was further classified as the early-onset (E-PE) or late-onset (L-PE) [35, 36] subtype. Considering the much earlier gestational age of E-PE placentas, the placentas from the patients with unexplained preterm labor (PTL) served as the gestational-week matched controls for E-PE according to previous reports [15]. Unexplained PTL was defined as labor of unknown causes earlier than the 34th week but without any other diagnosable pregnancy problems. Pregnancies complicated by gestational diabetes, hypertensive disorders, renal or cardiovascular disease, intrauterine fetal death, fetal chromosomal or congenital abnormalities, or pregnancies that were conceived with the assistance of reproductive technologies were excluded. In total, placental tissues from patients with PTL (n = 7), E-PE (n = 16), normal pregnancy (CTRL, n = 14) and L-PE (n = 10) were included in this study. The tissues were subjected to protein or RNA extraction, paraffin embedding or flash freezing in liquid nitrogen within 1 h of the surgery. The clinical characteristics of the enrolled pregnant women are summarized in Additional file 1: Table S1.

### Cell culture and treatment

The immortalized human trophoblast cell line HTR8/SVneo was kindly provided by Dr. C.H. Graham at Queen’s University, Canada. The cells were cultured in Roswell Park Memorial Institute 1640 (RPMI 1640) medium (Invitrogen, California, USA) supplemented with 10% fetal bovine serum (FBS; GIBCO, New York, USA). HTR8/SVneo cells were starved for 24 h in media containing 0.5% FBS before treatment with HGF (R&D Systems, Minnesota, USA) at various concentrations. HEK293T cells were purchased from American Type Culture Collection (ATCC, USA) and cultured in Dulbecco’s modified Eagle’s medium (DMEM) (Invitrogen, California, USA) supplemented with 10% FBS.

Transient transfection experiments were carried out by using Lipofectamine 2000 (Invitrogen, California, USA). The siRNA duplexes were synthesized by GenePharma Co., Ltd. (Shanghai, China), and the sequences were as follows: si-Met (sense, 5′-GCCUGAAUGAUGACAUUCUTT-3′; antisense, 5′-AGAAUGUCAUCAUUCAGGCTT-3′), si-CAV-1 (sense, 5′-CCCACUCUUUGAAGCUGUUTT-3′; antisense, 5′-AACAGCUUCAAAGAGUGGGTT-3′), si-Cbl (sense, 5′-GGCGAAACCUAACCAAACUTT-3′; antisense, 5′-AUGGGGGUUAGGUUUCGCCTT-3′) and scramble negative control (sense, 5′-UUCUCCGAACGUGUCACGUTT-3′; antisense, 5′-ACGUGACACGUUCGGAGAATT-3′).

### Generation of plasmids carrying Met cDNA

The full-length human Met coding sequence (cDNA) was amplified and inserted into the pcDNA3.1 vector (Invitrogen, California, USA) to generate the Met^WT^ expression plasmid. The Met mutant Met^Y1003F^ was constructed by using the QuickChange site-directed mutagenesis kit (Stratagene, California, USA), with the tyrosine^1003^ residue being mutated to a phenylalanine residue. The primers for mutagenesis were 5′-GGAAAAGTAGCTCGGAAGTCTACAGATTCATTTGAAACCATTTCT-3′ (forward) and 5′-AGAAATGGTTTCAAATGAATCTGTAGACTTCCGAGCTACTTTTCC-3′ (reverse). The sequences were verified by DNA sequencing (Invitrogen, California, USA) and then ligated into the pcDNA3.1 vector.

### RNA preparation and quantitative real-time polymerase chain reaction (PCR)

Total RNA was extracted with TRIzol reagent (Invitrogen, California, USA) and reverse transcribed into cDNA using Oligo (dT) primers (Amersham Biosciences, USA). Quantitative real-time PCR was carried out by using a LightCycler 480 (Roche, Basel, CH). The primer sequences were as follows: human Met, 5′-TGCCCAGACCCCTTATATGAAG-3′ (forward) and 5′-GATATCCGGGACACCAGTTCAG-3′ (reverse); human CAV-1, 5′-TTCGCCATTCTCTCTTTCCT-3′ (forward) and 5′-CAGCTTCAAAGAGTGGGTCA-3′ (reverse); human clathrin, 5′-CACAATGCCTTAGCCAAAATC-3′ (forward) and 5′-CGACGTACCAGGTAGCGAGAA-3′ (reverse); human Cbl, 5′-ACCACGACTTGACCTTCTGC-3′ (forward) and 5′-GTCGGGATTCTGCTCCAACA-3′ (reverse); human glyceraldehyde-3-phosphate dehydrogenase (GAPDH), 5′-GAAGGTGAAGGTCGGAGTC-3′ (forward) and 5′-GAAGATGGTGATGGGATTTC-3′ (reverse); and human beta-2-microglobulin (B2M), 5′-TGGGTTTCATCCATCCGACA-3′ (forward) and 5′-ACATGTCTCGATCCCACTTAAC-3′ (reverse). All PCRs were performed in triplicate, and the relative expression levels were determined by the 2^−ΔΔCT^ method and normalized to GAPDH or B2M [37].

### Western blot analysis

Total proteins from the placental tissues or the cultured cells were extracted with radioimmunoprecipitation assay (RIPA) lysis buffer, and 40 μg protein of each sample was subjected to 10% sodium dodecyl sulfate-polyacrylamide gel electrophoresis (SDS-PAGE) and subsequent electrotransfer onto nitrocellulose membranes (Amersham Pharmacia Biotech, Buckinghamshire, UK). The membranes were further incubated with the indicated antibodies (Additional file 1: Table S2) followed by horseradish peroxidase (HRP)-conjugated secondary antibody (Promega, Wisconsin, USA). Signal visualization was achieved by ECL reagents and an Enhanced Chemiluminescence Western Blotting System (Pierce, Illinois, USA). The relative density of each specific molecule was determined by normalization to that of β-actin in the same blot.

### Transmission electron microscopy (TEM)

Five placentas from each group (PTL, E-PE, CTRL and L-PE) were subjected to the TEM assay. Biopsied placenta (1 mm^3^) tissues were freshly collected from five different sites located 5 cm from the umbilical cord insertion and fixed in 3% glutaraldehyde overnight. After rinsing and fixation in 1% osmium tetroxide, the samples were dehydrated and embedded in an epon-araldite mixture. Semithin sections at 0.6 μm were prepared and stained with uranyl acetate and lead citrate, followed by examination with a JEM-1400 (JEOL, JAPAN) transmission electron microscope.

### Immunofluorescence staining and confocal microscopy

Freshly collected tissues were embedded in Tissue-Tek optimal cutting temperature (O.C.T) compound (Sakura Finetek, USA). Frozen sections (8 μm thick) were incubated with primary antibodies (Additional file 1: Table S2) and visualized using a fluorescein isothiocyanate (FITC)- or tetraethyl rhodamine isothiocyanate (TRITC)-conjugated secondary antibody (Zhongshan Goldenbridge, China) and 4′,6-diamidino-2-phenylindole (DAPI) (Sigma-Aldrich, Missouri, USA). The negative control was set by replacing the primary antibody with preimmune immunoglobulin G (IgG) (Zhongshan Golden Bridge, China). The signals were captured by an LSM780 laser confocal microscope (Zeiss, Oberkochen, Germany). Imaris software was used to quantitatively analyze the colocalization of Met and the proteasome.

### Biotinylation internalization and recycling assays

Measurement of the proportions of internalized or recycled Met was performed as described previously with slight modification [38]. In brief, the cultured cells were incubated with 0.2 mg/ml Sulfo-NHS-SS-Biotin (Pierce, Illinois, USA) on ice to label cell-surface proteins. After washing with cold phosphate-buffered saline (PBS) to remove unlabeled biotin, the cells were further incubated in culture media at 37 °C for 4 h to allow protein trafficking, and the biotin remaining at the cell surface was subsequently removed using the membrane-impermeant reducing agent sodium 2-mercaptoethane sulfonate at 180 mM (MesNa, Sigma-Aldrich, Missouri, USA) in Tris buffer at pH 8.6. Following the quenching of MesNa by iodoacetamide (IAA, Sigma-Aldrich, Missouri, USA), the cells were lysed, and the extracted proteins were incubated with streptavidin-agarose beads to enrich biotin-labeled proteins for Western blotting.

To measure the recycling of internalized Met, the cells were subjected to a 4 h incubation in media at 37 °C after the aforementioned MesNa treatment. After that, the second round of cell-surface biotin reduction with MesNa and quenching of MesNa with IAA was performed, and the cell lysates were processed for Western blotting.

Two controls were set in each internalization or recycling assay. (1) Cell lysates were collected just after biotin labeling of cell-surface proteins (time 0) to measure total surface Met. (2) The cells were subjected to MesNa treatment immediately after biotin labeling of cell-surface proteins (time 0), and the cell lysates were used to test the efficiency of surface biotin removal.

Equivalent volumes of cell lysates were subjected to Western blotting for Met, densitometric analyses were performed, and Met internalization or recycling was calculated as follows. Index of Met internalization = (Met level following incubation at 37 °C for 4 h − Met level at time 0)/(total surface Met) × 100. Index of Met recycling = 100 − [(Met level at 4 h of reincubation at 37 °C − Met level at time 0)/(Met level at 4 h of the first incubation at 37 °C − Met level at time 0) × 100].

### Transwell invasion assay

In vitro cell invasion was assayed by determining the ability of cells to invade a synthetic basement membrane, a growth factor-reduced Matrigel matrix (BD Biosciences, New Jersey, USA). The experiment was carried out as described previously [39]. All experiments were performed in triplicate, and the invasion index is presented as the percentage of invaded cells compared with the corresponding control.

### Statistical analysis

All statistical analyses were performed with the Statistical Program for Social Sciences (SPSS) 18.0 software package (IBM). Differences between groups were analyzed using the two-tailed Student’s t test or one-way analysis of variance (ANOVA) (for normally distributed data), followed by post hoc Tukey–Kramer multiple comparison analysis. Differences were considered significant at P < 0.05. Data are shown as the mean ± standard error of the mean (SEM) based on three independent experiments.

## Results

### Punctate aggregation of Met and increased endocytic vesicles in the placentas of subjects with E-PE

**Fig. 1:**
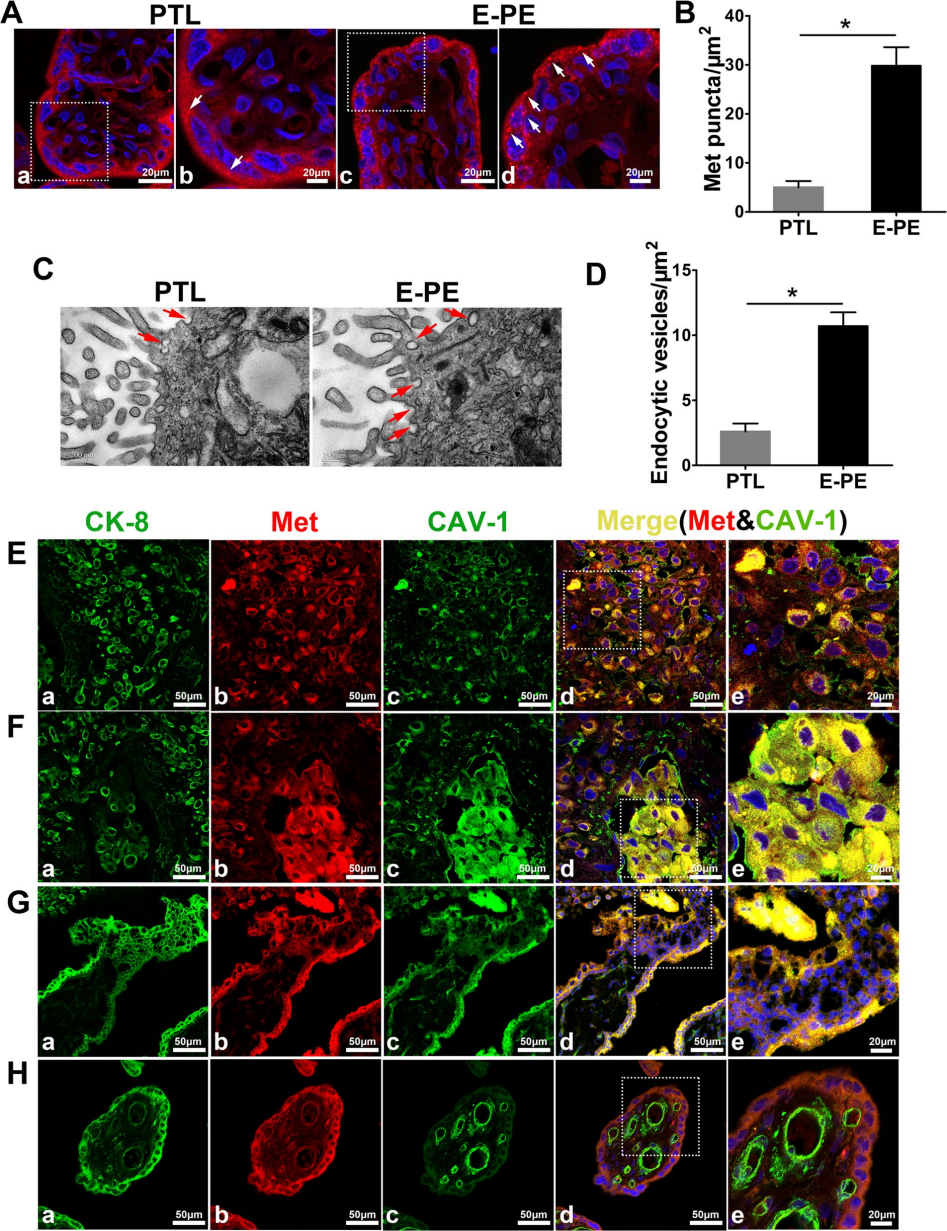
Distribution of Met and CAV-1 in placentas derived from E-PE and PTL. **A** Typical results of high-resolution immunofluorescence analysis of Met in gestational age-matched placentas of patients with PTL and E-PE. b and d, Higher magnification images of a and c, respectively. The arrows indicate punctate aggregates (50–100 nm) of Met. Scale bar, 20 μm. **B** Bar chart showing the statistical analysis of the number of Met punctate aggregates in trophoblasts per μm^2^ of the placenta. **C** Representative electron microscopy images of syncytiotrophoblasts in PTL and E-PE placentas. The arrows indicate the endocytic vesicles on the cell surface. Scale bars, 200 nm. **D** Bar chart showing the statistical results of the average number of endocytic vesicles per μm^2^ of the cell. The statistical analyses in B and D were performed based on the data from 5 placentas per group and 10 random visual fields in each placenta section. **E**–**H** Immunofluorescence assay of CK8 (a), Met  (b) and CAV-1 (c) in normal first-trimester decidual (**E** and **F**), villous tissues (**G**), and term placental tissues (**H**). Met and CAV-1 were stained in the same section, and CK8 was stained in the adjacent section to identify trophoblast cells. Panel e in (**E**–**H**) shows the higher magnification of the indicated area in panel d. Scale bars, a-d, 50 μm, e, 20 μm. The data are expressed as the mean ± SEM, and comparisons between the two groups were made by the two-tailed t test. *p < 0.05

Given that Met/Erk signaling was inhibited in E-PE placentas [15], we analyzed the localization of Met in PE placentas using a high-resolution immunofluorescence assay to examine pathological changes in Met. The results showed obvious accumulation of intracellular Met punctate aggregates, with diameters of 50–100 nm, in the trophoblast cells of E-PE placentas. However, these signals were less intense in the placentas from the PTL, L-PE and CTRL groups (Fig. 1A, Additional file 1: Fig. S1A, B). Quantitative statistical analysis revealed that the number of Met punctate aggregates in the E-PE trophoblasts was 5.4-fold higher than that in the PTL controls (Fig. 1B). In parallel, TEM observation and the corresponding quantitative statistics showed more endocytic vesicles on the trophoblastic cell membrane from the E-PE placentas than the PTL controls (Fig. 1C, D). However, there was no difference between the L-PE and CTRL groups (Additional file 1: Fig. S1C, D). Such aberrant distribution of Met and endocytic vesicles in E-PE placenta suggested that the abnormal endocytosis and/or degradation processes of Met may occur in E-PE trophoblasts.

### Colocalization of Met and CAV-1 in human placental trophoblast cells

The endocytosis of membrane proteins in mammalian cells is mediated by mainly Clathrin and CAV-1-coupled caveolae [40–42]. To clarify the pathway via which Met endocytosis may occur, immunofluorescence analyses of Met and CAV-1 were carried out in placental villi and decidua at gestational weeks 7–8, with staining for cytokeratin 8 (CK8) in adjacent sections to mark trophoblast cells (Fig. 1E–H, a). Met was distributed in interstitial extravillous trophoblasts (iEVTs, Fig. 1E, b), endovascular extravillous trophoblast cells (enEVTs, Fig. 1F, b) and villous trophoblast cells (VTs, Fig. 1G, b). Costaining of punctate Met aggregates with CAV-1 was observed in some iEVTs, enEVTs and VTs (Fig. 1E–G, d e). In normal-term placenta, colocalization of Met with CAV-1 was observed in VTs and villous vascular endothelial cells (Fig. 1H, d e). However, the immunofluorescent signal for Clathrin was faint in the placental tissues, and little colocalization of Met and Clathrin was observed (Additional file 1: Fig. S2). These results indicated that Met endocytosis in trophoblasts may predominantly depend on the CAV-1-mediated pathway.

### The interaction of Met and CAV-1 is enhanced by hypoxia in trophoblastic cells

**Fig. 2:**
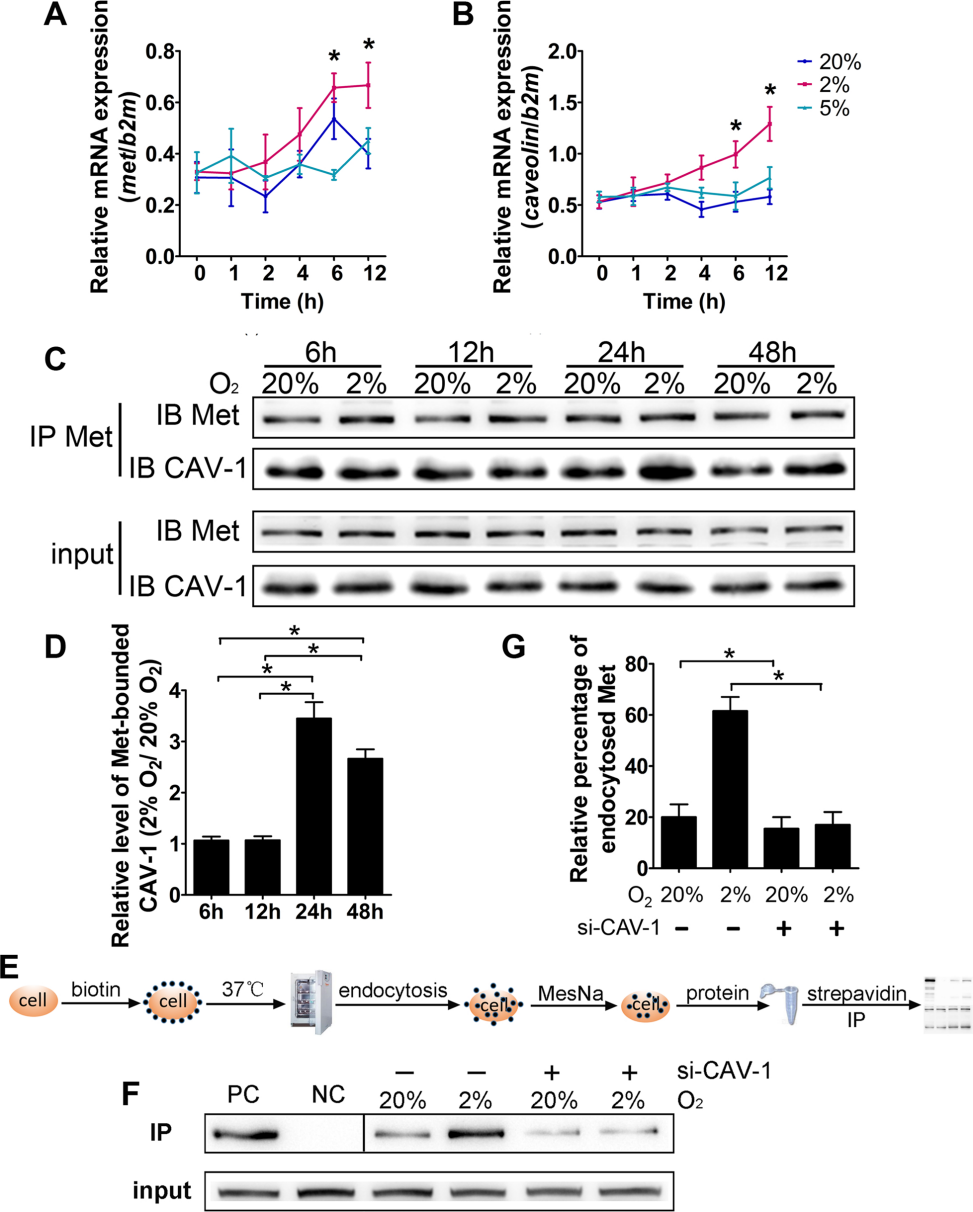
Binding of Met to CAV-1 and endocytosis of Met in HTR8/SVneo cells exposed to hypoxia. **A**–**B** Results of real-time quantitative PCR showing the expression of Met and CAV-1 in HTR8/SVneo cells that were exposed to various concentrations of O_2_ for 1 h to 12 h. **C**–**D** Typical results (**C**) and statistical results (**D**) of Co-IP showing the interaction of Met and CAV-1 in HTR8/SVneo cells that were exposed to 20% or 2% O_2_ for 6 h to 48 h. **E** Schematic diagram illustrating the procedure for the biotinylation internalization assay. Details are described in the Methods, and the small black dots indicate the biotin-labeled cell-surface proteins. **F**–**G** Typical results (**F**) and statistical results (**G**) of the biotinylation internalization assay of Met in HTR8/SVneo cells that were transfected with or without a specific siRNA for CAV-1 and exposed to 20% or 2% O_2_ for 4 h. All statistical analyses were performed based on the results of three independent experiments. The data are presented as the mean ± SEM, and comparisons between groups were analyzed by one-way ANOVA followed by the Tukey–Kramer multiple comparisons post hoc test. *Comparison between the indicated groups, p < 0.05

To further prove that the endocytosis of Met is mediated by CAV-1 in trophoblast cells, the interaction between Met and CAV-1 was examined in a trophoblastic cell line, HTR8/SVneo. Considering that hypoxic stress is well recognized to be a trigger for trophoblastic injuries in the PE placenta, we cultured HTR8/SVneo cells under 2% or 5% O_2_ to mimic the pathological hypoxia observed in the PE placenta [29–31]. Quantitative real-time PCR revealed upregulated levels of Met and caveolin (the gene encoding CAV-1) upon exposure to 2% O_2_ for 6–12 h compared with those in the corresponding normoxic control group (20% O_2_), whereas exposure to 5% O_2_ had little influence on the expression of Met and CAV-1 (Fig. 2A and B). However, the expression of clathrin in HTR8/SVneo cells was not changed after hypoxic stress exposure (Additional file 1: Fig. S3).

A coimmunoprecipitation (co-IP) experiment was carried out in these cells, and the binding between CAV-1 and Met was observed at various O_2_ concentrations (Fig. 2C and Additional file 1: Fig. S4A). In addition, the binding of CAV-1 to Met was significantly enhanced under sustained exposure to 2% O_2_ for 24–48 h (Fig. 2C, D), but Met expression was not influenced by 5% O_2_ exposure (Additional file 1: Fig. S4A, B). Furthermore, co-IP between Met and clathrin did not result in any positive band, suggesting that Met and clathrin do not interact in the cells (Additional file 1: Fig. S4C). These results confirmed that CAV-1 can effectively bind to Met in trophoblastic cells and that the binding is enhanced upon exposure to sustained severe hypoxia.

### Hypoxia triggers intensive CAV-1-dependent Met endocytosis in trophoblastic cells

To quantitively measure the endocytosis of Met, a biotinylation internalization assay was performed in HTR8/SVneo cells. In brief, the cell-surface proteins were labelled with sulfo-NHS-SS-biotin, and the cells were then incubated at 37 °C to allow protein trafficking. Cell-surface biotin was subsequently removed by using MesNa, and intracellular biotinylated Met was measured by streptavidin pulldown and Western blotting (Fig. 2E). A basic level of Met endocytosis was observed under normoxic conditions, and hypoxic exposure (2% O_2_) significantly boosted the endocytosis of Met (3.2-fold higher than that in the control group) (Fig. 2F, G). Furthermore, when CAV-1 was knocked down with a specific siRNA, the endocytosis-enhancing effect of hypoxia was abrogated in the cells (Fig. 2F, G).

The endocytosis and exocytosis of membrane receptors dynamically equilibrate physiological activities in mammalian cells [43]. We performed a biotinylation recycling assay to assess the exocytosis of Met (Additional file 1: Fig. S5 A) and found little difference in Met exocytosis under the conditions of 20% and 2% O_2_ (Additional file 1: Fig. S5B and C). The above data revealed that Met was endocytosed through CAV-1-coupled caveolae in trophoblasts and that the process was significantly boosted by hypoxic stress.

### Intracellular punctate aggregates of Met increasingly accumulate in parallel with the repressed expression of Cbl in trophoblasts under hypoxic stress conditions

**Fig. 3:**
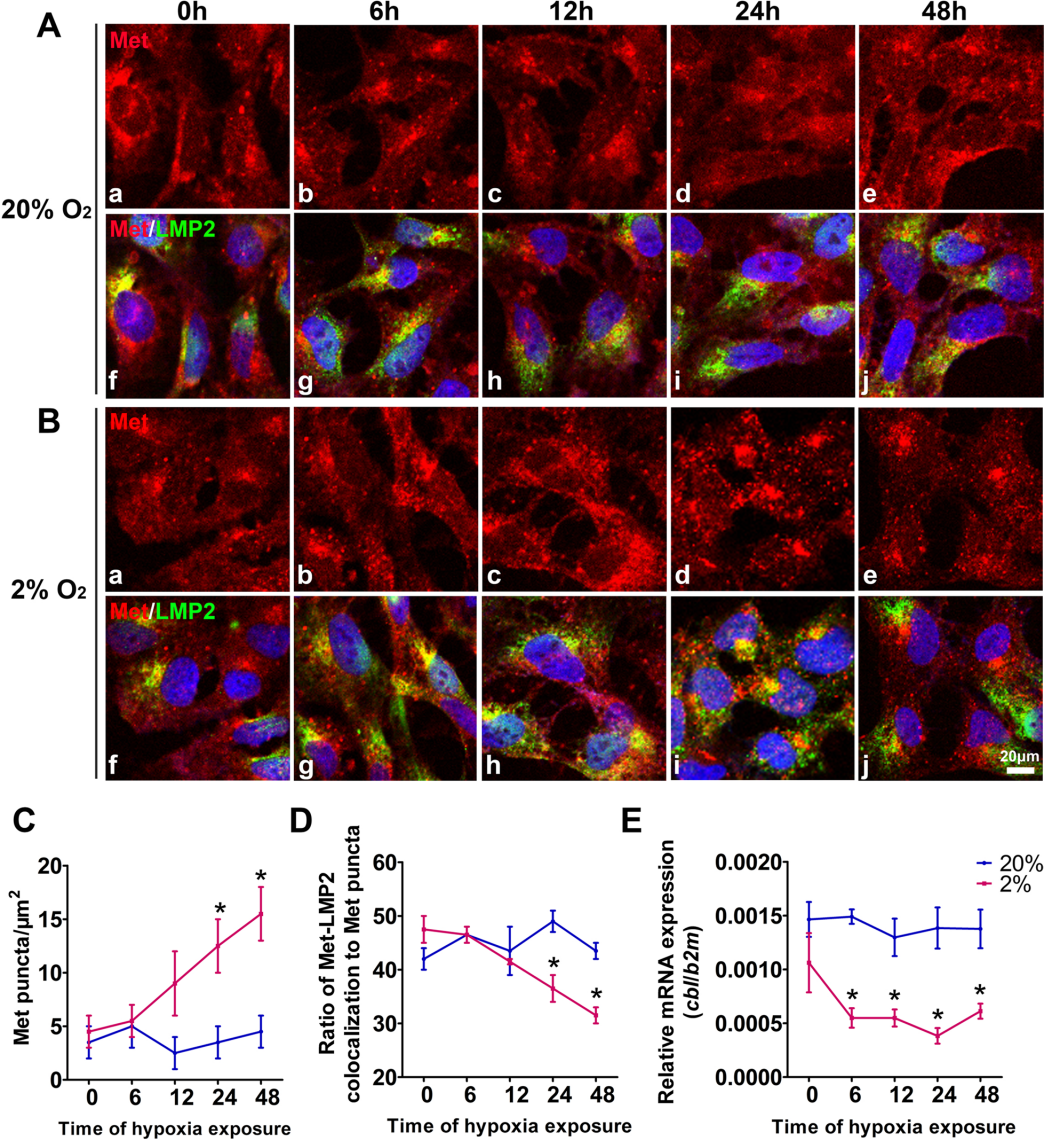
Colocalization of Met with the proteasome and the expression of Cbl in HTR8/SVneo cells under hypoxia. **A–B** Typical results of the high-resolution immunofluorescence analysis of Met (a–e) and Met/LMP2 (f–j) in HTR8/SVneo cells that were exposed to 20% or 2% O_2_ for 0 h to 48 h. Scale bar, 20 μm. **C** Quantitative statistics of Met punctate aggregation at the indicated times (A-B). Line chart showing the numbers of Met punctate aggregates per μm^2^ in HTR8/SVneo cells. **D** Line chart showing the ratio of Met-LMP2 colocalization to Met puncta in the cells, as determined by the statistical analysis of three sections per group and 5 random fields of view in each section. **E** Results of real-time quantitative PCR showing the expression of Cbl in HTR8/SVneo cells that were exposed to various concentrations of O_2_ for 1 h to 48 h. The data are expressed as the mean ± SEM and were analyzed by the two-tailed t test based on at least three independent experiments. *Compared with the corresponding data in the control, p < 0.05

To assess the influence of hypoxia on the distribution of Met in HTR8/SVneo cells, immunofluorescence was performed in the cells at various time points following 2% O_2_ exposure. As shown in Fig. 3A–B, a certain amount of cytoplasmic Met puncta was observed in HTR8/SVneo cells under 20% O_2_, which did not change during 0–48 h of culture. However, when the cells were exposed to 2% O_2_, the number of Met punctate aggregates in the cytoplasm increased time-dependently, exceeding those in the corresponding normoxic control at 24 and 48 h by 4.1- and 4.7-fold, respectively (Fig. 3A–C).

Such abundant accumulation of intracellular Met may also have been due to hampered ubiquitin degradation in the cells. To address this, Met and proteasomes (using an antibody against proteasome 20S LMP2) were double-stained, and the Met-LMP2 colocalization signal was quantitatively analyzed (Fig. 3A, B). The ratio of Met-LMP2 colocalization to the total intracellular Met signal in the prolonged hypoxia exposure group was significantly reduced by 47% compared to that in the normoxic control group at 24 and 48 h (Fig. 3D), leading to the substantial repression of Met proteolysis in the cells subjected to sustained hypoxia exposure.

Cbl is reported to be the specific ubiquitin protein ligase (E3) of Met that binds the phosphorylated tyrosine of its intracellular domain [30, 31, 44]. Our quantitative real-time PCR results showed decreased Cbl expression in hypoxia-exposed HTR8/SVneo cells from 6 to 48 h (Fig. 3E). These data suggested that the repressed ubiquitin degradation of Met may also contribute to its punctate aggregation in trophoblasts upon prolonged exposure to hypoxic stress.

### Cbl knockdown increases the punctate aggregation of Met in HTR8/SVneo cells

**Fig. 4:**
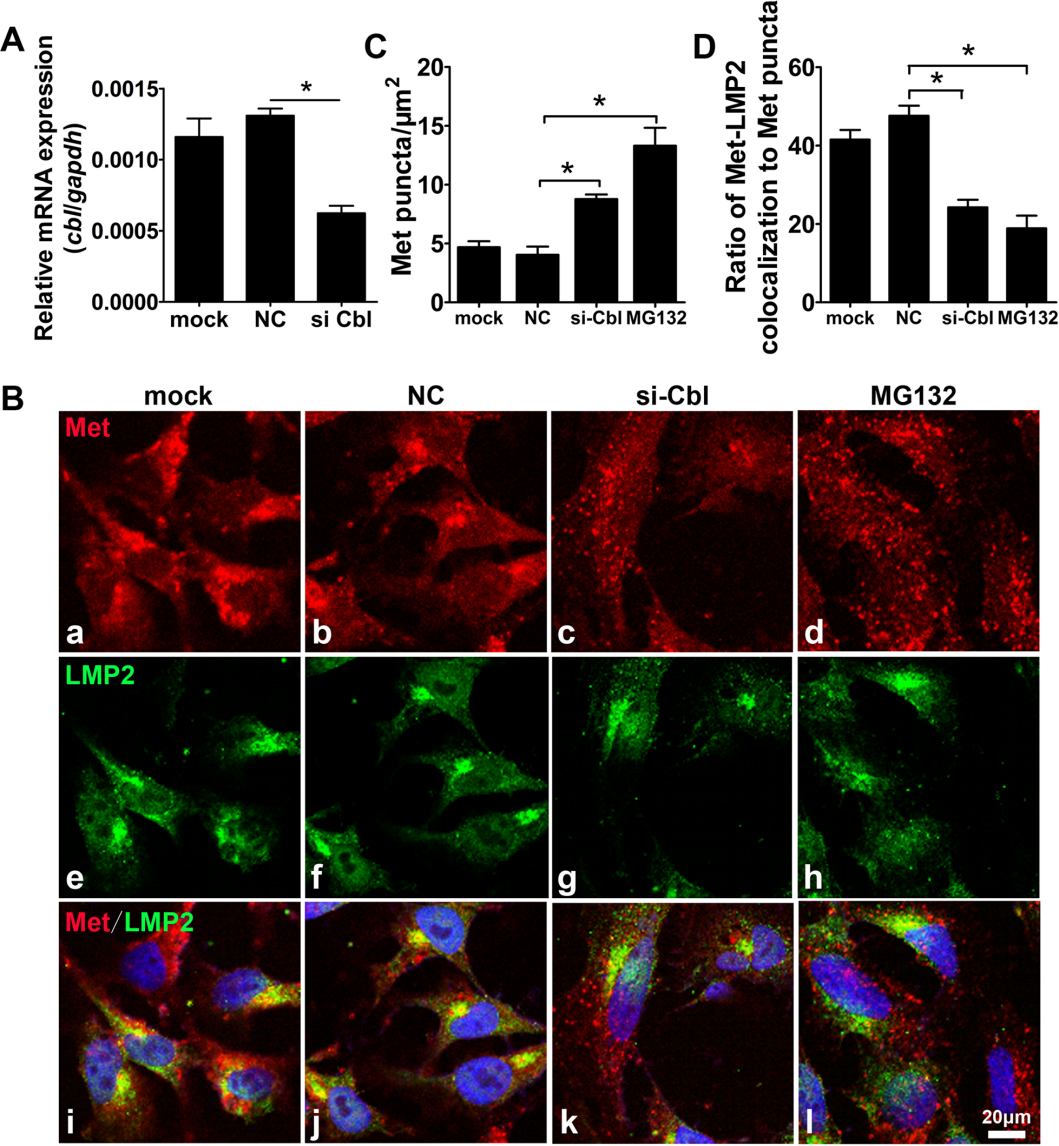
The distribution of Met aggregates and proteasomes in HTR8/SVneo cells upon Cbl knockdown. **A** Results of real-time quantitative PCR showing the expression of Cbl in HTR8/SVneo cells transiently transfected with a specific siRNA for Cbl (si-Cbl) or a scramble siRNA (NC) or exposed to only the transfection reagent (mock). **B** Typical results of the high-resolution immunofluorescence analysis of Met (a–d), LMP2 (e–h), and Met/LMP2 (i–l) in HTR8/SVneo cells transfected with NC, si-Cbl for 48 h or exposed to the proteasome inhibitor MG132 (5 μM). Scale bar, 20 μm. **C** Bar chart showing the numbers of Met punctate aggregates per μm^2^ in HTR8/SVneo cells. **D** Bar chart showing the ratio of Met-LMP2 colocalization to Met puncta in the cells, as determined by the statistical analysis of three sections per group and 5 random fields of view in each section. The data are presented as the mean ± SEM, and comparisons between groups and were analyzed by one-way ANOVA (for normally distributed data) followed by the Tukey–Kramer multiple comparisons post hoc test. *Compared with the corresponding data in the control, p < 0.05

To further confirm the role of ubiquitylation degradation in the hypoxia-induced aggregation of Met in trophoblast cells, we utilized an siRNA to knock down Cbl, the specific E3 ubiquitin ligase of Met, in HTR8/SVneo cells (Fig. 4A) and examined the distribution of Met by immunofluorescence. Cells treated with a proteasome inhibitor (MG132) were recruited as a positive control [45]. As shown in Fig. 4B and C Cbl knockdown enhanced the aggregation of Met in HTR8/SVneo cells, as the number of Met punctate aggregates was increased by twofold compared to that in the control, similar to MG132 exposure. Furthermore, double staining for Met and LMP2 revealed that the ratio of Met-proteosome colocalization was reduced by 50% in the Cbl knockdown and MG132-treated cells compared to the corresponding controls (Fig. 4B and D). These findings indicated that the downregulation of Cbl could hamper the degradation and thus the punctate intracellular aggregation of Met.

### Cbl knockdown suppresses the invasion-promoting effect of HGF in HTR8/SVneo cells

**Fig. 5:**
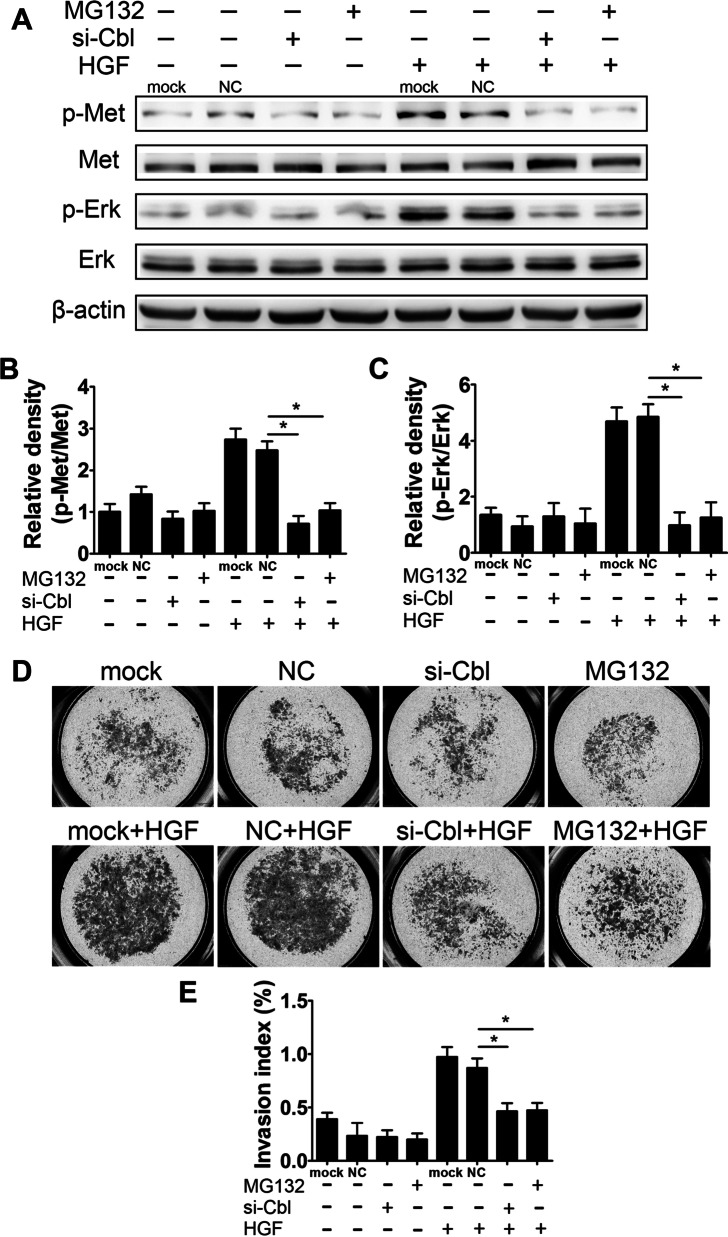
Effect of Cbl knockdown on HGF-induced Met activation and cell invasiveness in HTR8/SVneo cells. **A** Typical results of Western blotting showing the levels of p-Met, Met, p-Erk and Erk in HTR8/SVneo cells upon Cbl knockdown with or without HGF (20 ng/ml) and MG132 (5 μM) exposure. **B, C** Bar chart showing the relative densities of p-Met/Met and p-Erk/Met. **D** Transwell invasion assay of HTR8/SVneo cells upon Cbl knockdown with or without 20 ng/ml HGF exposure. **E** Quantitative statistics of the percentage of invaded cells compared with the corresponding control. The data are presented as the mean ± SEM, and comparisons between groups were analyzed by one-way ANOVA followed by the Tukey–Kramer multiple comparisons post hoc test based on at least three independent experiments. *Compared with the corresponding data in the control, p < 0.05

To further clarify whether intracellular Met accumulation affects HGF/Met signaling, we examined HGF-stimulated Met activation and cell invasion following Cbl knockdown in HTR8/SVneo cells. Surprisingly, the HGF-induced activation of Met and downstream Erk was remarkably abrogated by Cbl knockdown or MG132 exposure, whereas the basal levels of phosphorylated Met and Erk were not affected (Fig. 5A–C). In parallel, HGF significantly promoted the cell invasion ability (increased by 2.5-fold compared to that in the control group), and the effect was completely abrogated by Cbl knockdown or MG132 exposure (Fig. 5D and E). Downregulation of Cbl slightly decreased the basal level of cell invasiveness, but the difference was not statistically significant (Fig. 5D and E).

To further confirm whether the binding of Cbl to Met affects the activation of Met, we generated a plasmid encoding ubiquitination-deficient Met (Met^Y1003F^), in which the Cbl binding site was mutated from tyrosine to phenylalanine [46]. The wild-type Met (Met^WT^) and Met^Y1003F^ plasmids were separately transfected into 293 T cells, in which the expression of endogenous Met was very low. Exposure of the cells to HGF resulted in the phosphorylation of Met; however, the level of phosphorylated Met in Met^Y1003F^-transfected cells was less than half of that in the Met^WT^ group (Additional file 1: Fig. S6). Collectively, these data reveal that Cbl-mediated Met ubiquitin degradation plays a vital role in maintaining the activity of HGF/Met signaling.

### Cbl and CAV-1 are aberrantly expressed in the placentas of patients with E-PE

**Fig. 6:**
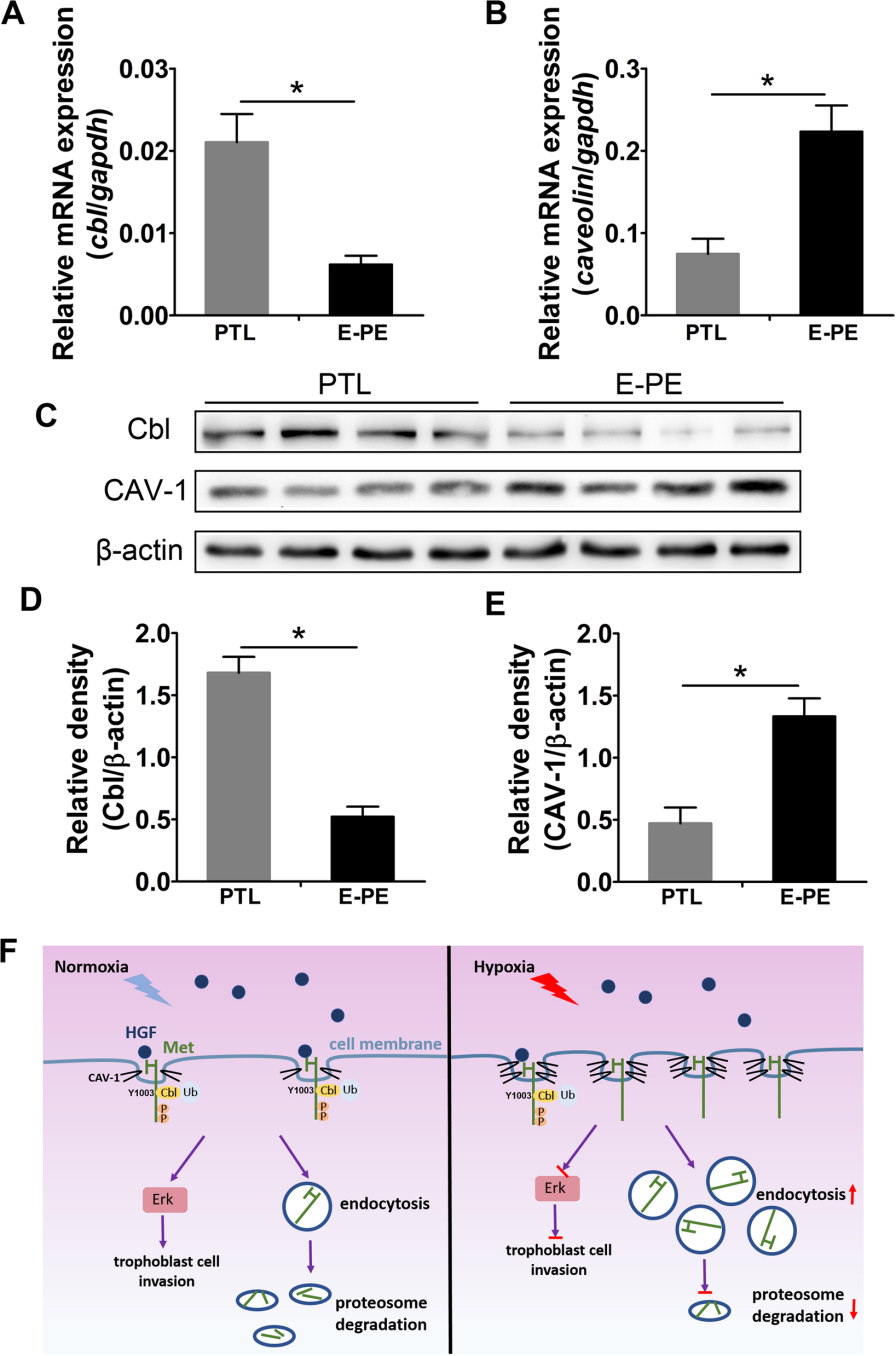
Expression of Cbl and CAV-1 in the placentas of patients with E-PE and PTL. **A, B** Results of real-time quantitative PCR showing the relative expression of Cbl and CAV-1 in the PTL (n = 7) and E-PE (n = 16) placentas. **C** Typical results of Western blotting showing the protein levels of Cbl and CAV-1 in the PTL (n = 7) and E-PE (n = 16) placentas. **D, E** Bar chart showing the relative densities of Cbl and CAV-1. **F** Working model illustrating the endocytosis and degradation of Met in trophoblasts under physiological normoxic and pathologically sustained hypoxic conditions. Under normoxic conditions (left panel), the binding of HGF to Met activates Met-Erk signaling to enhance trophoblast invasion and induce CAV-1-coupled endocytosis and the subsequent Cbl-mediated degradation of Met, thereby maintaining the homeostasis of HGF/Met signaling in placental trophoblasts. However, when trophoblasts face prolonged hypoxic stress (right panel), the augmented endocytosis via CAV-1 and hampered Cbl-mediated degradation of Met lead to the aberrant intracellular accumulation of Met and thus to the impaired activation of Met/Erk signaling and dysregulation of trophoblast differentiation. The data are expressed as the mean ± SEM and were analyzed by the two-tailed t test based on at least three independent experiments. *Compared with the corresponding data in the control, p < 0.05

The levels of Cbl and CAV-1 were measured in the PE and PTL placentas of subjects enrolled in this study. Both quantitative real-time PCR and Western blotting revealed significantly decreased expression of Cbl in the E-PE placentas. Compared with those in the PTL control, the RNA level was reduced by 68% and the protein level was reduced by 63% (Fig. 6A, C, D). The RNA level of CAV-1 was upregulated by 2.9-fold, and its protein expression was upregulated by 2.3-fold in the E-PE placentas compared with the PTL controls (Fig. 6B, C, E). However, the expression levels of Cbl and CAV-1 in L-PE placentas were not significantly different from those in the CTRL group (Additional file 1: Fig. S7).

## Discussion

Accumulating in vitro and in vivo evidence has revealed the importance of the HGF/Met signaling pathway for placental development, particularly in regulating trophoblast cell differentiation and endothelial tubulogenesis [10, 47]. Genetic deficiency of HGF or Met in mice causes severely impaired placentation and midgestational lethality [10, 31]. Our previous study demonstrated remarkably reduced activation of Met and downstream Erk signaling in the placentas of E-PE patients and suggested hampered transactivation of Met by Sema4D in PE trophoblasts [15]. Here, we further revealed abnormal endocytosis and proteosomal degradation of Met in E-PE placental trophoblasts, which led to the aberrant retardation of Met recycling and thus hindered HGF/Met signaling to regulate trophoblast invasion. These findings substantiate the contribution of compromised HGF/Met function to the occurrence of PE, especially the early-onset subtype; however, the expression of HGF and Met has not been shown to differ in PE placentas [48, 49].

The endocytosis/exocytosis and intracellular degradation of membrane receptors are important for maintaining the homeostasis of many growth factor-mediated signaling pathways [50–54]. An important finding in this study is the vicious effect of persistent hypoxic stress on the endocytosis and ubiquitination degradation of the Met receptor in human trophoblasts. As we summarize in Fig. 6F, the binding of HGF to the Met receptor triggers the phosphorylation of Met and the downstream Erk signal cascade and initiates the CAV-1-coupled endocytosis and Cbl-mediated ubiquitination degradation of Met. The physiological process ensures the transmission of HGF signals to an appropriate extent to control trophoblast differentiation. Pathologically prolonged hypoxia in trophoblast cells induces excessive Met endocytosis via CAV-1-coupled caveolae and hampers the proteosomal degradation of Met, together leading to the augmented intracellular accumulation of the Met protein and thus to the failure of HGF/Met signaling to modulate trophoblast invasiveness.

The placenta is critical for the health of the mother and the growing fetus during pregnancy [55]. As the major cell type in the placenta, one trophoblast cell subtype is invasive extravillous trophoblasts (EVTs), which can invade the uterine stroma and remodel uterine spiral arteries [56]. Appropriate uterine-placental-fetal circulation is established in this way to meet the requirements for maternal–fetal nutrient exchange. Shallow invasion of the uterine stroma and insufficient remodeling of spiral arteries by EVTs at early-to-mid gestation are well known as the predominant physiological changes leading to impaired blood perfusion into the placentas of PE patients [57]. Compromised trophoblast cell invasion due to dysregulated Met signaling may exacerbate placental hypoxia due to the limited remodeling of uterine spiral arteries by trophoblasts, thus forming a vicious feedback loop that may eventually lead to the occurrence of E-PE. Therefore, the findings in this study provide new evidence to at least partially elucidate the underlying molecular etiology of E-PE.

Our recent study performed using a mouse model proved that HGF/Met signaling participates in regulating trophoblast cell fate along multiple differentiation pathways [8]. Although we confirmed only that the blockage of HGF-promoted trophoblast invasion by hampering the ubiquitin degradation of Met in this study, it can be reasonably speculated that various trophoblast cell behaviors are dysregulated by hampered HGF/Met activity under sustained hypoxia. The accumulation of Met puncta in various trophoblast subtypes in the E-PE placenta may reflect the multifaceted defects in these cells that are caused by sustained severe hypoxia. HGF/Met is also an important angiogenetic signal that promotes endothelial tubulogenesis [58]. Colocalization of Met with CAV-1 was observed in the villous vascular endothelium, the branching of fetal blood vessels. Thus, further investigation is warranted to determine whether impaired Met recycling and activation occur in villous vessels of the E-PE placenta, which may adversely affect fetal development.

In addition to Met, hypoxia may damage the recycling of other membrane receptors in the E-PE placenta. Although little is known about the placenta in this regard, evidence from other cells or systems may provide some hints. In *C. elegans*, hypoxia results in the internalization of GLR-1 and thus the reduction of glutamate-activated currents and the depression of GLR-1-mediated neuronal sensing [26]. Rapid downregulation of the surface transferrin receptor by oxidative stress represents the physiological mechanism of reduced iron uptake in diverse pathological conditions, including hypoxia-reperfusion injury, acquired immunodeficiency syndrome, and aging [59]. On the other hand, evidence has confirmed the association of dysregulated ubiquitin-related factors with the onset of PE. For instance, the constitutive photomorphogenic-9 (COP9) signalosome (CSN) complex forms an important part of the ubiquitin proteasome system (UPS) that is involved in modulating signal transduction, gene transcription, and protein stability in trophoblast cells [60]. The upregulation of CSN1 and CSN5 may be associated with the pathophysiology of PE [60]. The scaffold protein Cullin 3 (CUL3) participates in the assembly of numerous ubiquitin ligase complexes. Silencing CUL3 significantly disturbs the invasion and migration of trophoblast cells [61]. Therefore, in-depth exploration of these abnormalities in receptor recycling in the PE placenta may help to clarify the compromised regulatory signals of the disease.

It has been suggested that E-PE and L-PE may have different causes [62]. E-PE is more likely to arise from the malfunctioning placenta under hypoxic and oxidative stress, especially via the inadequate blood perfusion through uterine spiral arteries. However, L-PE appears to be influenced by environmental and maternal nutrition factors during the late stages of gestation [27, 28, 32]. Studies by ourselves and others performed using mouse models have shown the predominant role of HGF/Met signaling in regulating the early differentiation fate of trophoblasts [8, 10, 33, 63]. Aberrantly repressed Met/Erk signaling due to the hindered recycling of Met upon prolonged exposure to hypoxia was identified in E-PE placental trophoblasts but not in L-PE placentas. All this evidence further supports that E-PE and L-PE originate from various pathological factors.

There are some limitations in this study. The differentiation and functional maintenance of trophoblast cells at the maternal–fetal interface involve dynamic interactions among multiple cell types and complicated regulatory networks comprising multiple factors and signaling pathways. In addition, a variety of pathological causes, such as hypoxia, inflammation, and metabolic disorders, are multifactorially associated with PE. Therefore, appropriate in vivo models are indispensable for fully clarify the contribution of repressed Met activity to adverse pregnancy outcomes. Trophoblast-specific silencing of Cbl or overexpression of CAV-1 may help to address this point. Moreover, other signaling pathways that may interrupt Met activation need to be further explored. These findings may provide insights into possible therapeutic interventions for PE, especially the early-onset subtype.

## Conclusions

Our study highlights the pathological mechanism of Met signaling inhibition in the E-PE placenta. Sustained hypoxia causes excessive CAV-1-coupled endocytosis and suppression of the Cbl-induced ubiquitin degradation of Met, disrupting the HGF/Met regulation of trophoblast cell invasion. The findings deepen our understanding of the molecular basis of abnormal trophoblast differentiation in adverse pregnancy outcomes and may further the development of therapeutic approaches for E-PE.

## Supplementary Information

**Additional file 1: Table S1.** Clinical characteristics of the women enrolled in this study. **Table S2.** Antibodies used in this study. **Figure S1.** The distribution of Met and endocytic vesicles in the CTRL and L-PE placentas. A, Typical results of high-resolution immunofluorescence for Met in gestational age-matched placentas of CTRL and L-PE. The arrows indicate punctate aggregates of Met. Scale bar, 20 μm. B, Bar chart showing the statistical analysis of the number of Met punctate aggregates in trophoblasts per μm^2^ of the placenta. C, Representative electron microscopy images of syncytiotrophoblasts in CTRL or L-PE placentas. The arrows indicate the endocytic vesicles on the cell surface. Scale bars, 200 nm. D, Bar chart showing the statistical results of the average number of endocytic vesicles per μm^2^ of the cell. The statistical analysis in B and D was performed based on the data from 5 placentas per group and 10 random visual fields in each placenta section. The data are expressed as the mean ± SEM, and comparisons between the two groups were made by the two-tailed t test. *, p<0.05. **Figure S2.** Localization of Met and clathrin in decidual and villous tissues of pregnant villi and placental tissues. A-D, Immunofluorescence assay for CK8, Met and clathrin in normal first-trimester villous (A), decidual tissues (B-C), and term placental tissues (D). Met and Clathrin were stained in the same section, and CK8 was stained in the adjacent section to identify trophoblast cells. In A and B, panel e shows the higher magnification of the indicated area in panel d. In C and D, panel d shows the higher magnification of the indicated area in panel c. E, panel a shows Clathrin-positive SH-SY5Y cells. Panel b is the negative control set obtained by replacing the primary antibody with IgG. **Figure S3.** Clathrin RNA expression under various oxygen concentrations. The results of real-time quantitative PCR showing the expression of Met and Clathrin in HTR8/SVneo cells that were exposed to various concentrations of O_2_ for 0 h to 12 h. The data are presented as the mean ± SEM, and comparisons between groups were analyzed by one-way ANOVA followed by the Tukey–Kramer multiple comparisons post hoc test based on at least three independent experiments. **Figure S4.** Interaction of Met and clathrin in HTR8/SVneo cells under different oxygen concentrations. A-B, Typical results (A) and statistical results (B) of Co-IP showing the interaction of Met and clathrin in HTR8/SVneo cells that were exposed to 20% or 5% O2 for 6 h to 48 h. C, Co-IP showing the interactions of Met, CAV-1 and clathrin. Cells were cultured in 2% O_2_ and 20% O_2_ for 6 h. The data are presented as the mean ± SEM, and comparisons between groups were made by one-way ANOVA followed by the Tukey–Kramer multiple comparisons post hoc test based on at least three independent experiments. **Figure S5.** Exocytosis of Met in HTR8/SVneo cells under different oxygen concentrations. A, Schematic diagram of the biotinylation recycling assay. Details are described in the Methods, and the small black dots indicate the biotin-labeled cell-surface proteins. B, Typical results of the biotinylation recycling assay. HTR8/SVneo cells were cultured at 20% O_2_ or 2% O_2_. The levels of surface biotinylated Met that were internalized (4 h) and recycled (4 h) were measured. IP showed the level of Met after streptavidin pulldown. C, Bar chart showing the relative level of recycled Met. Index of Met recycling = 100- [(Met level at 4 h of reincubation at 37°C - Met level at time 0)/(Met level at 4 h of the first incubation at 37°C - Met level at time 0)×100]. The data are expressed as the mean ± SEM, and analysis was carried out by the two-tailed t test based on at least three independent experiments. **Figure S6.** MetY1003F overexpression attenuates Met activation in 293T cells. A, Typical results of Western blotting showing the levels of p-Met and Met in 293T cells upon transfection with wild-type MetWT or mutant MetY1003F (2 μg) and with or without HGF (20 ng/ml) exposure. B, Bar chart showing the relative density of p-Met/Met. The data are presented as the mean ± SEM, and comparisons between groups were made by one-way ANOVA followed by the Tukey–Kramer multiple comparisons post hoc test based on at least three independent experiments. *, compared with corresponding data in the control, p<0.05. **Figure S7. **The expression of Cbl and CAV-1 in CTRL and L-PE placentas. A-B, Results of real-time quantitative PCR showing the relative expression of Cbl and CAV-1 in the CTRL (n=14) and L-PE placentas (n=10). C, Typical results of Western blotting showing the protein levels of Cbl and CAV-1 in the CTRL (n=14) and L-PE placentas (n=10). D-E, Bar chart showing the relative density of Cbl and CAV-1. The data are expressed as the mean ± SEM, and analysis was carried out by the two-tailed t test based on at least three independent experiments.

## Data Availability

The datasets used and/or analyzed during the current study are available from the corresponding author on reasonable request.
