# Supplementary material for: Hypoxic stress disrupts HGF/Met signaling in human trophoblasts: implications for the pathogenesis of preeclampsia
Source: J Biomed Sci. 2022 Feb 3;29:8. doi: 10.1186/s12929-022-00791-5 (PMC8815204; doi:10.1186/s12929-022-00791-5)
Supplement: Supplementary file 1 — Additional file 1: Table S1. Clinical characteristics of the women enrolled in this study. Table S2. Antibodies used in this study. Figure S1. The distribution of Met and endocytic vesicles in the CTRL and L-PE placentas. A, Typical results of high-resolution immunofluorescence for Met in gestational age-matched placentas of CTRL and L-PE. The arrows indicate punctate aggregates of Met. Scale bar, 20 μm. B, Bar chart showing the statistical analysis of the number of Met punctate aggregates in trophoblasts per μm2 of the placenta. C, Representative electron microscopy images of syncytiotrophoblasts in CTRL or L-PE placentas. The arrows indicate the endocytic vesicles on the cell surface. Scale bars, 200 nm. D, Bar chart showing the statistical results of the average number of endocytic vesicles per μm2 of the cell. The statistical analysis in B and D was performed based on the data from 5 placentas per group and 10 random visual fields in each placenta section. The data are expressed as the mean ± SEM, and comparisons between the two groups were made by the two-tailed t test. *, p<0.05. Figure S2. Localization of Met and clathrin in decidual and villous tissues of pregnant villi and placental tissues. A-D, Immunofluorescence assay for CK8, Met and clathrin in normal first-trimester villous (A), decidual tissues (B-C), and term placental tissues (D). Met and Clathrin were stained in the same section, and CK8 was stained in the adjacent section to identify trophoblast cells. In A and B, panel e shows the higher magnification of the indicated area in panel d. In C and D, panel d shows the higher magnification of the indicated area in panel c. E, panel a shows Clathrin-positive SH-SY5Y cells. Panel b is the negative control set obtained by replacing the primary antibody with IgG. Figure S3. Clathrin RNA expression under various oxygen concentrations. The results of real-time quantitative PCR showing the expression of Met and Clathrin in HTR8/SVneo cells that were exposed [file 12929_2022_791_MOESM1_ESM.docx]

# DATA SUPPLEMENT

**Hypoxic stress disrupts HGF/Met signaling in human trophoblasts: implications for the pathogenesis of preeclampsia**

Guanlin Li^#^, Yongqing Wang^#^, Guangming Cao^#^, Yeling Ma, Yu-Xia Li, Yangyu Zhao*, Xuan Shao*, Yan-Ling Wang*

## Table S1. Clinical characteristics of the women enrolled in this study.

| **Characteristics** | **PTL**  **(n=7)** | **E-PE**  **(n=16)** | **CTRL**  **(n=14)** | **L-PE**  **(n=10)** |
| --- | --- | --- | --- | --- |
| Maternal age (yr) | 30.3±0.9 | 31.7±1.5 | 31.6±0.7 | 29.4±1.2 |
| BMI (kg/m^2^) | 22.9±0.6 | 24.2±1.8 | 22.3±1.1 | 24.7±0.8 |
| SBP (mm Hg) | 109.3±5.6 | 175.4±6.2* | 114.3±2.2 | 163.4±4.9^#^ |
| DBP (mm Hg) | 73.1±4.4 | 110.9±5.8* | 74.5±1.9 | 108.6±3.7^#^ |
| 24-h proteinuria (g/24 h) | ND | 4.3±0.4 | ND | 4.1±0.6 |
| Gestational age at delivery (d) | 228.3±7.7 | 231.6±5.9 | 275.3±5.4 | 276.6±7.8 |

BMI, body mass index; SBP, systolic blood pressure; DBP, diastolic blood pressure; ND, not determined; PTL, preterm labor; E-PE, early-onset preeclampsia; CTRL, control; L-PE, late-onset preeclampsia. Data are expressed as the mean±SEM, and comparisons between groups were analyzed by one-way ANOVA. *P<0.05 (compared with PTL), and ^#^ P<0.05 (compared with CTRL)

## Table S2. Antibodies used in this study.

| **Targets** | **Source** | **Brand** | **Product No.** | **Dilution** |
| --- | --- | --- | --- | --- |
| p-Met | Rb | CST | 3077 | WB1:1000 |
| p44/42 MAPK (Erk) | Mo | CST | 4696 | WB1:1000 |
| p- p44/42 MAPK (Erk) (Thr202/Tyr204) | Mo | CST | 9106 | WB1:1000 |
| Cbl | Rb | CST | 2747 | WB1:1000 |
| CD31 | Mo | CST | 3528 | IF1:500 |
| Met | Rb | Santa Cruz | sc-161 | WB1:1000, IP1:100, IF1:500 |
| Clathrin | Mo | Santa Cruz | sc-12734 | WB1:1000, IF1:500 |
| β-actin | Mo | Invitrogen | MA5-11869 | WB1:5000 |
| CK8 | Rb | Novus | NB100-79928 | IF1:5000 |
| LMP2 | Rb | Abcam | 3328 | IF1:500 |
| CAV-1 | Mo | Abcam | 17052 | WB1:1000, IF1:500 |

CST: Cell Signaling Technology, Massachusetts, USA. Santa Cruz: Santa Cruz Biotechnology, California, USA. Invitrogen: California, USA. Novus: Colorado, USA. Abcam: Cambridge, UK. Rb: Rabbit. Mo: Mouse. WB: Western blot. IF: immunofluorescence staining. IP: immunoprecipitation.

##


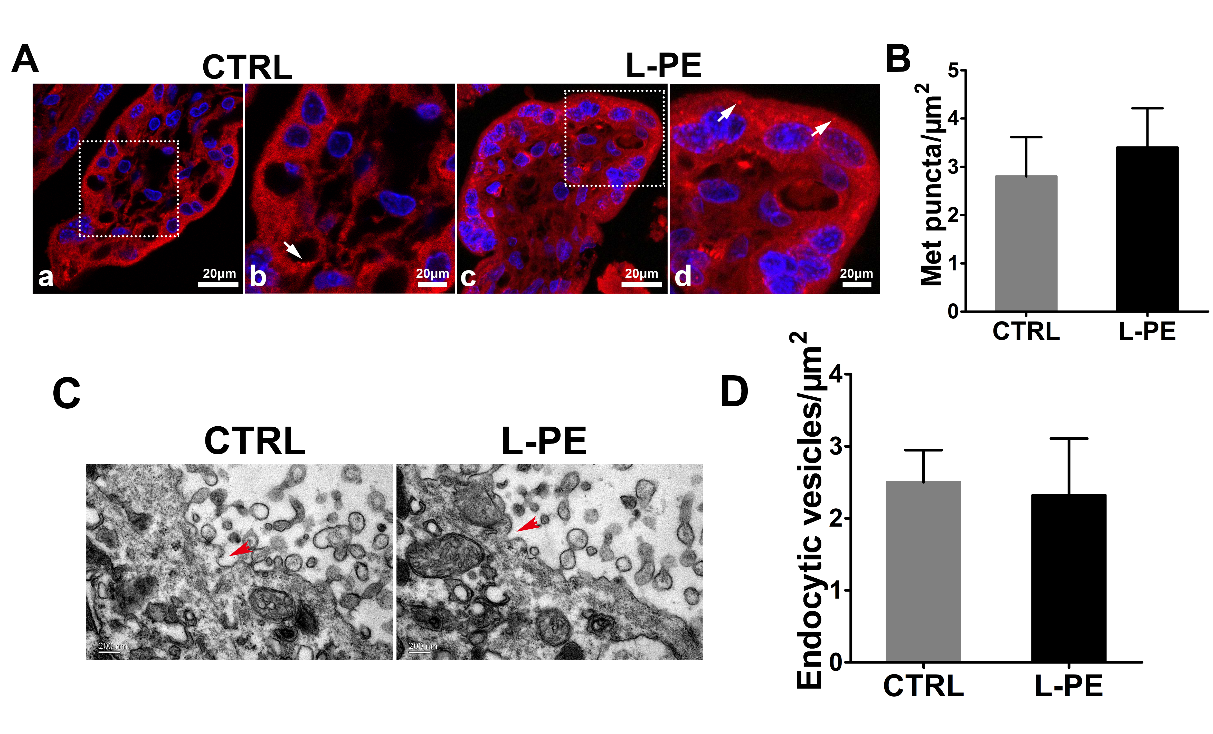


## Figure S1. The distribution of Met and endocytic vesicles in the CTRL and L-PE placentas. A, Typical results of high-resolution immunofluorescence for Met in gestational age-matched placentas of CTRL and L-PE. The arrows indicate punctate aggregates of Met. Scale bar, 20 μm. B, Bar chart showing the statistical analysis of the number of Met punctate aggregates in trophoblasts per μm^2^ of the placenta. C, Representative electron microscopy images of syncytiotrophoblasts in CTRL or L-PE placentas. The arrows indicate the endocytic vesicles on the cell surface. Scale bars, 200 nm. D, Bar chart showing the statistical results of the average number of endocytic vesicles per μm^2^ of the cell. The statistical analysis in B and D was performed based on the data from 5 placentas per group and 10 random visual fields in each placenta section. The data are expressed as the mean ± SEM, and comparisons between the two groups were made by the two-tailed t test. *, p<0.05.


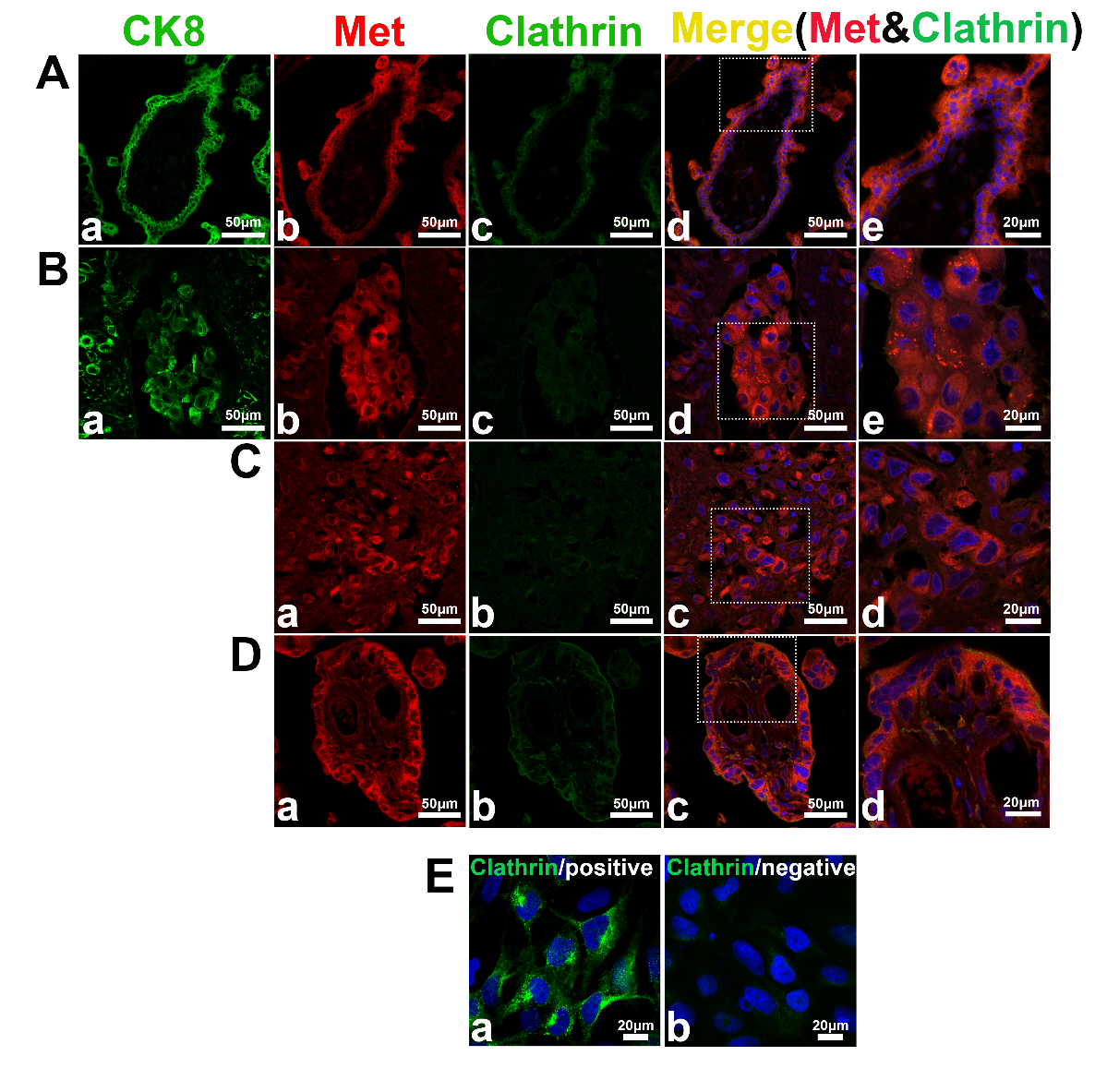


## Figure S2. Localization of Met and clathrin in decidual and villous tissues of pregnant villi and placental tissues. A-D, Immunofluorescence assay for CK8, Met and clathrin in normal first-trimester villous (A), decidual tissues (B-C), and term placental tissues (D). Met and Clathrin were stained in the same section, and CK8 was stained in the adjacent section to identify trophoblast cells. In A and B, panel e shows the higher magnification of the indicated area in panel d. In C and D, panel d shows the higher magnification of the indicated area in panel c. E, panel a shows Clathrin-positive SH-SY5Y cells. Panel b is the negative control set obtained by replacing the primary antibody with IgG.


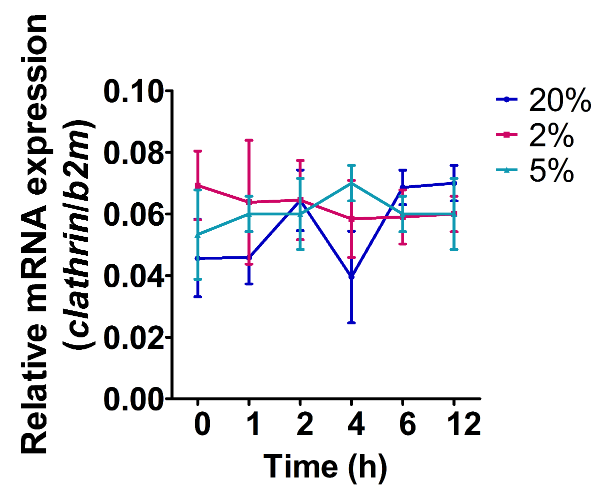


## Figure S3. Clathrin RNA expression under various oxygen concentrations. The results of real-time quantitative PCR showing the expression of Met and Clathrin in HTR8/SVneo cells that were exposed to various concentrations of O_2_ for 0 h to 12 h. The data are presented as the mean ± SEM, and comparisons between groups were analyzed by one-way ANOVA followed by the Tukey–Kramer multiple comparisons post hoc test based on at least three independent experiments.

##


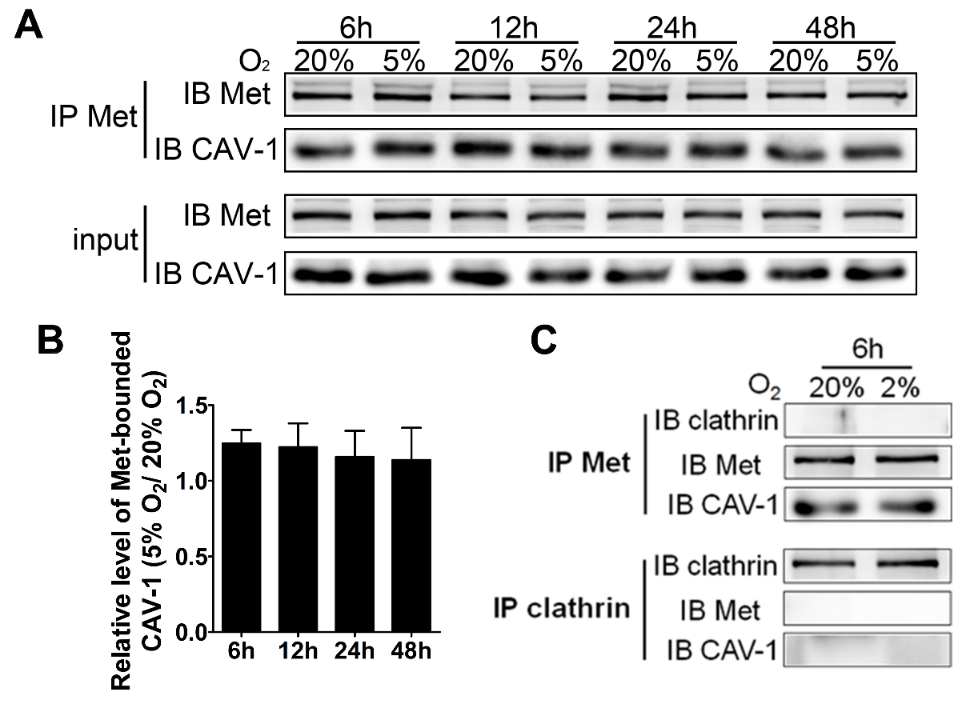


## Figure S4. Interaction of Met and clathrin in HTR8/SVneo cells under different oxygen concentrations. A-B, Typical results (A) and statistical results (B) of Co-IP showing the interaction of Met and clathrin in HTR8/SVneo cells that were exposed to 20% or 5% O_2_ for 6 h to 48 h. C, Co-IP showing the interactions of Met, CAV-1 and clathrin. Cells were cultured in 2% O_2_ and 20% O_2_ for 6 h. The data are presented as the mean ± SEM, and comparisons between groups were made by one-way ANOVA followed by the Tukey–Kramer multiple comparisons post hoc test based on at least three independent experiments.


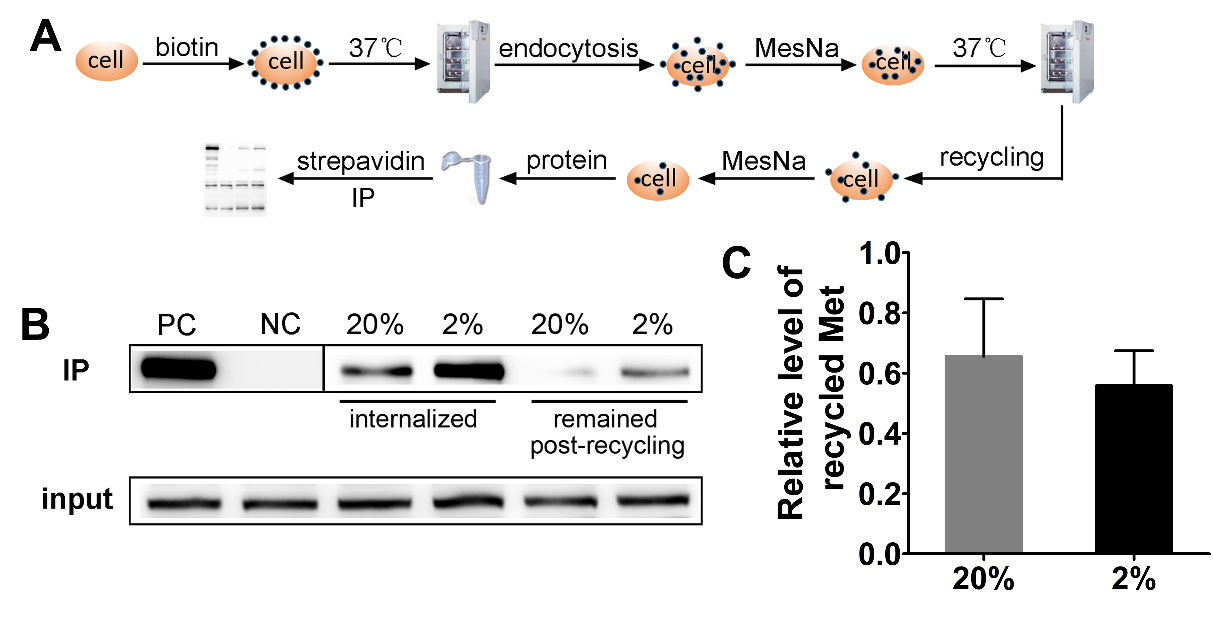


## Figure S5. Exocytosis of Met in HTR8/SVneo cells under different oxygen concentrations. A, Schematic diagram of the biotinylation recycling assay. Details are described in the Methods, and the small black dots indicate the biotin-labeled cell-surface proteins. B, Typical results of the biotinylation recycling assay. HTR8/SVneo cells were cultured at 20% O_2_ or 2% O_2_. The levels of surface biotinylated Met that were internalized (4 h) and recycled (4 h) were measured. IP showed the level of Met after streptavidin pulldown. C, Bar chart showing the relative level of recycled Met. Index of Met recycling = 100- [(Met level at 4 h of reincubation at 37°C - Met level at time 0)/(Met level at 4 h of the first incubation at 37°C - Met level at time 0)×100]. The data are expressed as the mean ± SEM, and analysis was carried out by the two-tailed t test based on at least three independent experiments.


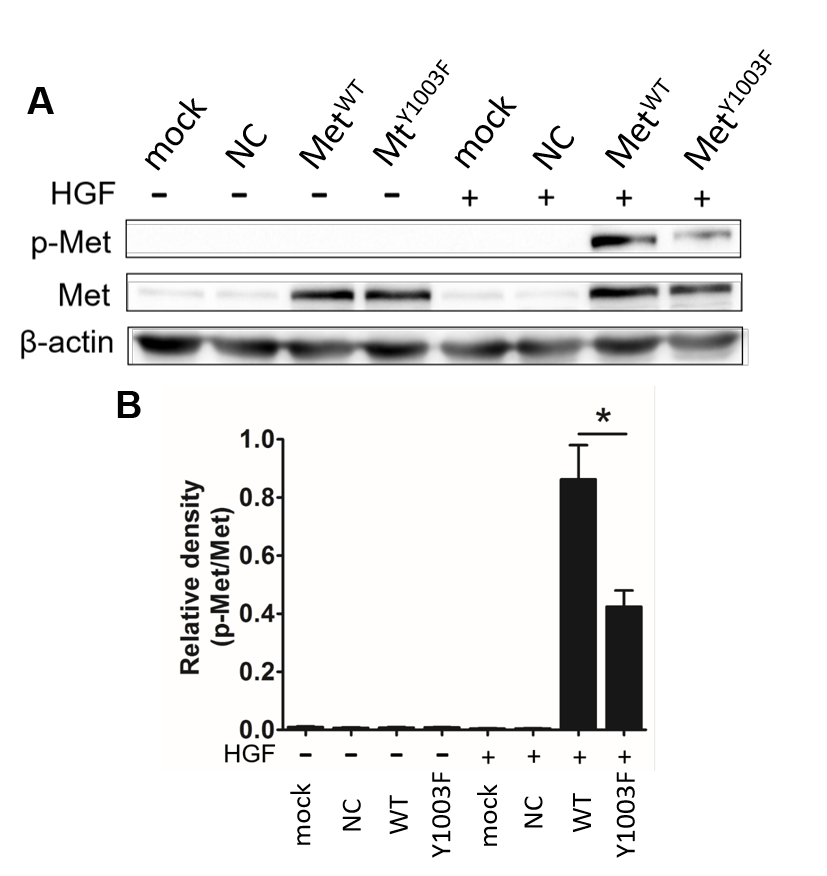


## Figure S6. Met^Y1003F^ overexpression attenuates Met activation in 293T cells. A, Typical results of Western blotting showing the levels of p-Met and Met in 293T cells upon transfection with wild-type Met^WT^ or mutant Met^Y1003F^ (2 μg) and with or without HGF (20 ng/ml) exposure. B, Bar chart showing the relative density of p-Met/Met. The data are presented as the mean ± SEM, and comparisons between groups were made by one-way ANOVA followed by the Tukey–Kramer multiple comparisons post hoc test based on at least three independent experiments. *, compared with corresponding data in the control, p<0.05.


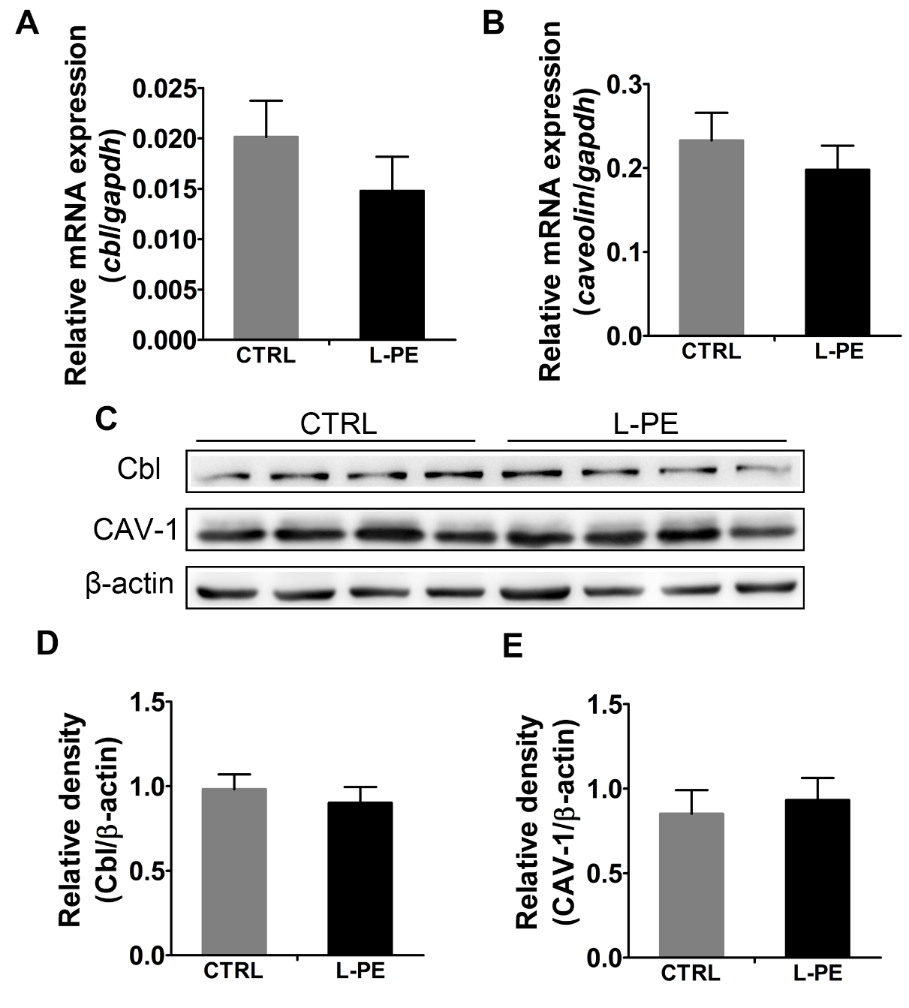


## Figure S7. The expression of Cbl and CAV-1 in CTRL and L-PE placentas. A-B, Results of real-time quantitative PCR showing the relative expression of Cbl and CAV-1 in the CTRL (n=14) and L-PE placentas (n=10). C, Typical results of Western blotting showing the protein levels of Cbl and CAV-1 in the CTRL (n=14) and L-PE placentas (n=10). D-E, Bar chart showing the relative density of Cbl and CAV-1. The data are expressed as the mean ± SEM, and analysis was carried out by the two-tailed t test based on at least three independent experiments.
